# Virtual craniotomy for high-resolution optoacoustic brain microscopy

**DOI:** 10.1038/s41598-017-18857-y

**Published:** 2018-01-23

**Authors:** Héctor Estrada, Xiao Huang, Johannes Rebling, Michael Zwack, Sven Gottschalk, Daniel Razansky

**Affiliations:** 1 Institute for Biological and Medical Imaging (IBMI), Helmholtz Center Munich, Neuherberg, Germany; 2 School of Medicine and School of Bioengineering, Technical University of Munich, Munich, Germany

## Abstract

Ultrasound-mediated transcranial images of the brain often suffer from acoustic distortions produced by the skull bone. In high-resolution optoacoustic microscopy, the skull-induced acoustic aberrations are known to impair image resolution and contrast, further skewing the location and intensity of the different absorbing structures. We present a virtual craniotomy deconvolution algorithm based on an ultrasound wave propagation model that corrects for the skull-induced distortions in optically-resolved optoacoustic transcranial microscopy data. The method takes advantage of the geometrical and spectral information of a pulse-echo ultrasound image of the skull simultaneously acquired by our multimodal imaging system. Transcranial mouse brain imaging experiments confirmed the ability to accurately account for the signal amplitude decay, temporal delay and pulse broadening introduced by the rodent’s skull. Our study is the first to demonstrate skull-corrected transcranial optoacoustic imaging *in vivo*.

## Introduction

From the multitude of techniques at our disposal for the quest to understand brain’s functionality^1^, optoacoustics has arguably become a promising tool for studying disease-related vascular changes^2^, rapid imaging of cerebral hemodynamics^3^, and monitoring neural activity via genetically-expressed indicators^4^. Yet all minimally-invasive neuroimaging and therapeutic techniques relying on transcranial ultrasound (US) transmission share a common problem, namely, acoustic distortions produced by the skull bone. Similarly to what one experiences when talking through a wall or a closed window, the transmitted acoustic intensity decays substantially, whereas the losses are more pronounced for shorter wavelengths^5,6^. Moreover, due to the curvature and heterogeneity of the skull bone, phase aberrations are additionally introduced into the ultrasonic wavefront. To this end, most of the efforts to correct for the skull-induced aberrations have been carried out in the context of High Intensity Focused Ultrasound (HIFU) brain therapy^7^. Simulations of US wave propagation through the whole human skull based on X-Ray Computed Tomographic (CT) scans using three-dimensional finite difference time domain calculations^8^ or reciprocal space methods^9,10^ are currently used in the clinical trials of HIFU brain therapy^11^.

Corrections for skull distortions are not commonly attempted in lower resolution optoacoustic (OA) systems used for whole-brain tomography^12,13^, chiefly because the effectively detected bandwidth corresponds to ultrasonic wavelengths that are much longer than the thickness of rodent skulls^14^, making the skull-induced distortions less significant. Approaches initially developed for HIFU, which solely make use of an average longitudinal speed of sound inferred from X-ray CT scans^15^, were attempted for correcting optoacoustic images of a monkey brain phantom via time reversal^16^. However, some of the underlying modelling assumptions in the HIFU case^11^ are not suitable for correcting high-resolution transcranial optoacoustic microscopy (OAM) scans for two main reasons: (1) as opposed to the narrowband continuous-wave acoustic excitation used in HIFU, the ultrawideband OA sources are localized not only in space but also in time; (2) HIFU is targeted onto a specific focal region inside the brain, while OA sources are distributed across the entire brain. As a result, the problem of transcranial OAM with high spatial resolution represents a much more comprehensive modeling challenge that also necessitates consideration of the shear wave components^17–20^. To accurately correct for the distortions produced by the passage of US waves originated in the mouse brain^6^, coregistered pulse-echo US images are ideally required for complementary structural information^21–24^.

In this work, we investigated the typical skull-induced distortions in transcranial OAM. An efficient virtual craniotomy (deconvolution) approach is then proposed, capable of recovering the original optoacoustically-generated data as if there was no skull in the US propagation path. In this way, we were able to obtain an accurate map of the skull position and infer its geometrical and mechanical properties, such as thickness, orientation, density and speed of sound, all of those entangled in the frequency spectrum of the reflected US wave. The algorithm, based on a rigorous three-dimensional acoustic wave propagation model further involves plane wave expansion to account for diffraction and refraction. Experimental validation is first performed with a point absorber (microsphere) and then applied to optically-resolved *in vivo* transcranial OAM mouse brain data.

## Results

**Figure 1:**
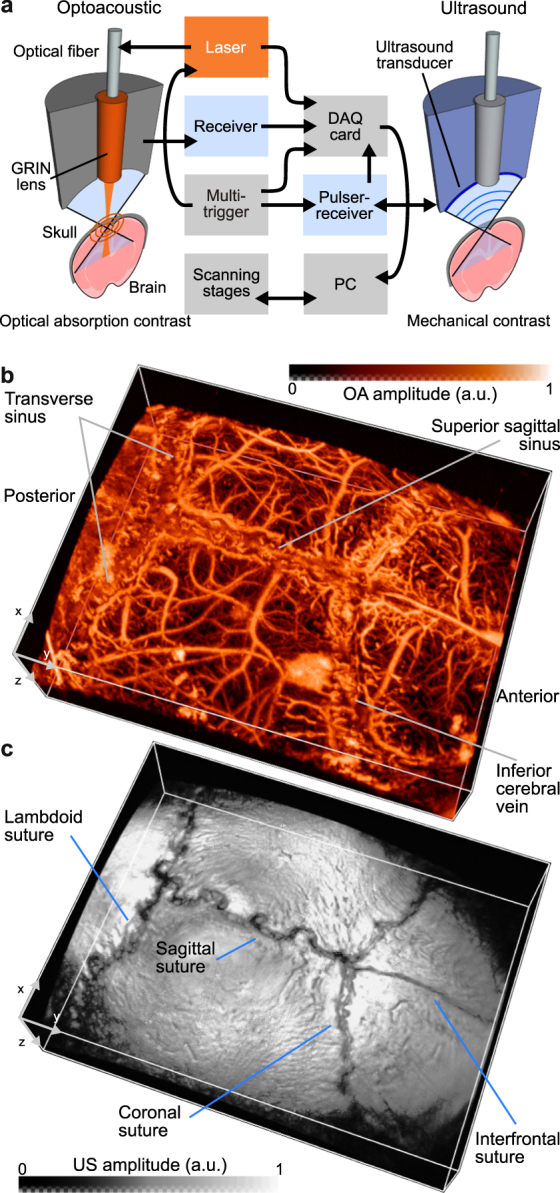
Dual mode optoacoustic and ultrasonic biomicroscopy of a mouse brain. (**a**) Diagram of the experimental setup featuring the hybrid imaging scanning head. Typical 3D optoacoustic and ultrasound datasets acquired form a mouse brain *in vivo* are shown in (**b**) and (**c**), respectively. No vesselness filter has been used to enhance the images.

**Figure 2:**
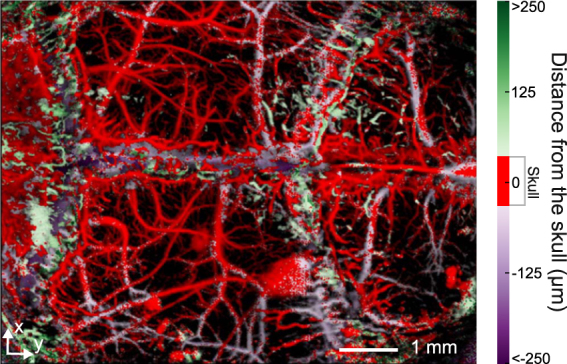
Maximum amplitude projection of the 3D mouse brain data obtained in the optical-resolution OA microscopy mode. The colormap represents distance of the signal’s maximum amplitude from the middle of the skull. Three regions can be distiguished: above (green), inside (red), and below (purple) the skull. No vesselness filter has been used to enhance the images.

Our dual-mode OA-US biomicroscope acquires the co-registered volumetric images of the skull and the brain using sequential excitation of the tissue with a pulsed laser source and an US pulser-receiver (Fig. 1). During the experiments, the excitation light wavelength was set to 578 nm while US detection and transmission was performed with a spherically focused detector that was additionally scanned for the 3D data acquisition (see Methods for details of the experimental setup). A closer analysis of the OA data readily reveals that the majority of the visible vasculature appears to be located inside the skull bone, as can be seen in the image color-coded for depth (Fig. 2). This obviously contradicts the common knowledge on the mouse anatomy and represents a major disadvantage of the imaging method since intracranial vasculature may be confused with vessels inside or on top of the skull^25^. The discrepancy can be attributed to reduction in the time-of-flight caused by the higher speed of sound in the skull as compared with the soft brain tissues and the coupling medium surrounding the skull. In addition, skull’s presence is known to introduce signal dispersion, which is responsible for a significant drop in the detected pressure magnitude and pulse broadening^6^.

**Figure 3:**
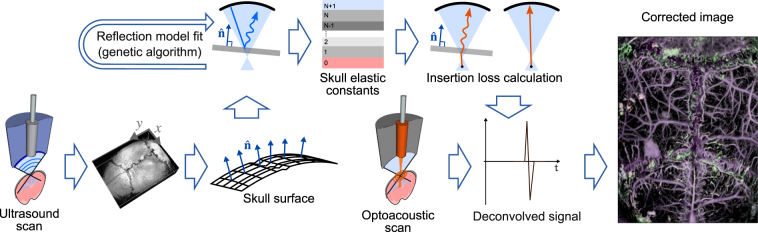
Workflow of the virtual craniotomy deconvolution algorithm. Transcranial 3D OA and pulse-echo US images are simultaneously acquired. The geometry of the skull and the apparent position of the brain vasculature are extracted using the time of flight and a constant speed of sound assumptions. The OA and US signals are then transformed into the frequency domain and the ultrasound reflection spectrum is fitted using a wave model. Subsequently, the insertion loss is calculated using the skull elastic constants that reached the best fit into the reflection model. The insertion loss is employed to deconvolve the OA signal spectrum. Finally, the deconvolved spectrum is transformed back into time domain and a distortion-corrected image of the mouse brain is formed.

The developed Virtual Craniotomy Algorithm (VCA) takes advantage of the geometrical and spectral information delivered by the pulse-echo US scans to correct for the skull’s distortions (Fig. 3). After scanning the mouse head with the two imaging modalities, the geometrical features of the skull and the vasculature are extracted from the time-domain data. OA signals located above the skull are excluded from the correction algorithm. Both OA and US data are then transformed into frequency domain. The frequency spectrum of the reflected US wave is used as a reference for a wave propagation model, which takes into account the transducer’s geometry and simulates the skull as a multilayered solid plate (see Supplementary information for details of the modeling methodology). A genetic algorithm^18^ optimizes the elastic constants of the solid and its thickness to fit the experimental data. The wave propagation model yields the insertion loss (IL)^18^ of the plate for a single point source excitation using the elastic constants that had the best fit to the reflection model. The IL is then used to deconvolve the transcranial OA signal. This procedure can be performed independently for each detected OA waveform provided that the OA wave resembles a single point source. In our case, the point source can be accurately mimicked if the lateral resolution of the images is governed by the diffraction-limited size of the focused light beam used for excitation of the OA signals.

**Figure 4:**
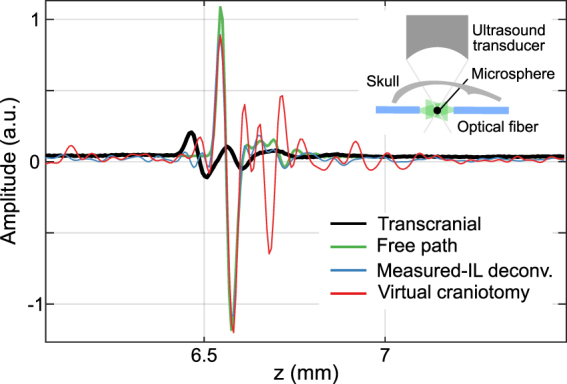
Calibration phantom experiments performed using a microsphere absorber, as shown in the inset. The optoacoustically generated signal is measured with and without the skull’s presence. The IL is calculated and used to recover the originally generated signal as if there was no skull present in the wave propagation path. Results of the band-limited IL deconvolution (best possible) and the VCA are shown together with the measured data.

We first tested the VCA in a phantom experiment designed to directly measure the IL^18^. A 20 µm absorbing sphere was illuminated using multimode optical fibers and the OA signals recorded with and without the skull present. To compare the VCA output against ideal propagation conditions, we deconvolved typical transcranially-detected signal with the measured IL under the same conditions as in the ideal wave propagation model (Fig. 4). One may readily notice the strong decrease in the signal amplitude, broadening of the wave packet, and the earlier signal arrival for the transcranial detection versus free-path propagation^6^. The VCA is able to recover the amplitude and the delay that match well characteristics of the free path signal. Yet, the VCA-corrected wave packet shows residual oscillations after the main bipolar pulse, which can be attributed to the imperfect fit of the skull´s elastic constants and the relatively large effective interaction area of the spherically focused detector with the skull surface.

**Figure 5:**
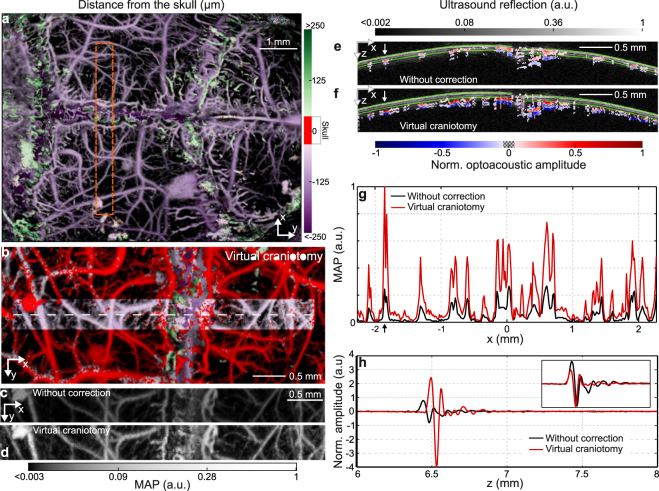
Results of the VCA correction applied to the *in vivo* transcranial imaging data. Depth-coded MAP image of the mouse brain vasculature data with (**a**) delay and (**b**) VCA correction. MAP images of the craniotomized region without (**c**) and with (**d**) VCA correction. A representative 2D cross-section (white dashed line in (**b**)) is shown for (**e**) uncorrected data and (**f**) after applying VCA. The B-mode ultrasound data are further shown for reference to delimit the skull boundaries (green lines). A relative 50% threshold has been applied to both uncorrected and corrected data for better visibility. (**g**) MAP of the cross section in (**e**) and (**f**). (**h**) Examples of single OA waveforms before and after the VCA correction. The signals correspond to the points indicated with an arrow in panels (**e**), (**f**), and (**g**). The inset in (**h**) shows a side-by-side comparison without relative delay and for the same signal amplitude. No vesselness filter has been used to enhance the images.

The VCA results for *in vivo* transcranial OA measurements are shown in Fig. 5. In contrast to the unprocessed images in Figs 2 and 5(e), where a large amount of vasculature appears to be located inside the skull bone, the VCA algorithm correctly assigns most of the vasculature to intracranial locations (Fig. 5(b,f)). Also, the signal amplitude is adjusted in accordance with the calculated IL (Fig. 5(c,d) and (g)). By inspecting an optoacoustic waveform (Fig. 5(h)), which was detected at a single detection point indicated by arrows in Fig. 5(e–g), one may note the corrected amplitude and delay, as well as the reduction in the skull-originated reverberations (inset in Fig. 5(h)). Indeed, the temporal profiles of the transcranial signals were shifted to their proper location and their amplitude restored without significantly affecting the noise level or the pulse width (Fig. 5(h)). Note that the algorithm has not altered the signals whose first positive peak originated above the skull surface. The latter correspond to some minor vasculature appearing in green color in Fig. 5(a and b).

## Discussion and Conclusions

Here we presented the first demonstration of skull-distortion correction for *in vivo* OA imaging. The proposed virtual craniotomy algorithm combines a fast deconvolution approach with a novel scheme to infer the elastic constants and geometry of the skull based on coregistered pulse-echo US data, all used for the first time in the context of correction for skull-induced acoustic aberrations.

The dual OA-US imaging capacity has been shown capable of discerning the correct location of the brain vasculature, which otherwise can only be blindly inferred when the additional pulse-echo US information is not available^3^. While simpler correction methods can be attempted, such as introducing temporal shifts based on the known speed of sound properties of the skull, those can merely compensate for the shift in the absorber location. Here, transcranial imaging experiments performed in living mouse brains confirmed the ability of VCA to accurately account for several skull-induced acoustic artifacts of major concern in OAM, including the signal amplitude decay, temporal delay, and pulse broadening, thus facilitating a better quantification of the size, location and strength of tissue absorbers.

The introduced correction methodology has yet several limitations. For analysis of the *in vivo* imaging results, the same average elastic constants were used across the entire skull. The constants as well as the average skull thickness were estimated at a single location where the normal to the skull surface coincides with the axis of the spherically focused transducer. Nonetheless, point by point characterization of those parameters is expected to provide even better results, at the expense of a significant increase in the computation time. Wave propagation techniques such as k-space^16^ or finite differences time domain^20^ methods could be used to further refine the accuracy of our results by including the inhomogeneous geometry of the skull, at the expense of a higher computational cost. Note that the sinus area is surrounded by hard tissue, thus the signals may arrive from above and within the sinus simultaneously. As a result, the correction accuracy is expected to be hampered in the areas close to the suture/sinus due to the higher modeling complexity.

The signal intensity amplification produced by the VCA largely depends on the size of the absorber (blood vessel) that generated the original signal. Since skull’s IL increases with frequency, the prevalent high frequency content generated by the small-sized capillaries is attenuated to a much greater extent as compared to signals from large vessels^5,18,26^. Thus, stronger amplification is generally expected for signals originating from smaller vessels.

Our IL estimations were based on a point source excitation, although the employed OAM has a long focal depth^27^ thus the excitation light beam may remain focused for a distance of 1 mm or more depending on tissue scattering. As a result, a quasi-cylindrical OA source may form within the imaged tissue with decaying amplitude along the axial (z) direction due to the light absorption by hemoglobin. The discrepancy between the point-source modeling assumption and realistic shape of the optical absorption source formed in tissue may affect accuracy of the VCA correction, especially with respect to the signal shape and width. In principle, the wave propagation model could be readily extended to simulate a line-shaped absorbing source at the expense of higher modeling and computational complexity. Likewise, the wave model may be further extended to account for arbitrary detection apertures, e.g. unfocused transducers or even multi-element arrays.

Age plays an important role in determining skull’s thickness, structure, and elastic properties. For instance, it is more likely that skulls of young mice mainly comprise of a cortical bone, thus the best VCA results were readily obtained by modeling with a single solid layer. In contrast, skulls of primates and humans have two cortical bone layers surrounding the trabecular bone^25^. In this case, successful adaptation of the VCA would likely imply the use of a multi-layered model while scaling down the frequency range to match the 1 cm-range skull thickness and the signal-to-noise performance of the experimental system.

From the modeling accuracy perspective, the effects of shear waves in a single fluid/solid interface should become significant for angles exceeding 20 degrees with respect to the skull’s normal^28^. The spherically-focused transducer used in the current experiments covers angles from −27 to 27 degrees. Thus, most of the shear wave components are expected to remain below the detection limit. Yet, previous experimental results^18^ show that the existence of a second solid/fluid interface (plate-like structure) may reduce the effective angle at which shear waves can be excited. In fact, the IL for a fluid-like skull (without considering shear waves) does not exhibit the same features as a solid skull; the former can be used to amplify and shift the OA signals to their original location, but cannot correct for pulse broadening. Thus, shear waves play a key role in reducing skull-induced reverberations.

Beyond the high-resolution microscopy applications in rodents, our universal modeling and correction framework is equally applicable to other implementations of transcranial OA and US imaging. Yet, it is anticipated that several parts of the wave propagation model can be refined to better grasp the physical nature of the related problems in larger animal models and, eventually, in human imaging applications, thus moving OA neuroimaging towards better and more quantitative images of the brain.

## Methods

### The hybrid optoacoustic and ultrasound biomicroscope

The experimental setup (Fig. 1(a)) has been previously reported and characterized^18,22^. Briefly, OA images are acquired by irradiating the mouse brain with 10 ns duration light pulses at 578 nm generated by a Dye laser system (Credo; Sirah Lasertechnik GmbH, Grevenbroich, Germany). The pulse repetition frequency is 10 kHz and each pulse carried an energy of 6 µJ. The light was guided through a single mode photonic crystal fiber (LMA20; NKT Photonics A/S, Birkerød, Denmark) and finally focused by a GRIN lens (Grintech GmbH, Jena, Germany) down to 20 µm diameter beam (full width at half maximum) impinging onto the tissue surface. Once the excitation light is absorbed by the brain tissue, the generated ultrasonic wave is detected by a spherically focused Polyvinylidene Fluoride (PVdF) ultrasound transducer (Precision Acoustics, Dorchester, United Kingdom) having a focal distance of 6.6 mm, 6 mm diameter aperture, 30 MHz center frequency, and 100% bandwidth. The transducer axis is aligned confocally with the illumination laser beam. The detected OA waveforms undergo a two-stage low-noise amplification with a total gain of 38 dB. Simultaneously, a photodiode is used to sample the per-pulse light energy in order to correct for the laser energy fluctuations.

The pulse-echo US image is produced by direct excitation of the transducer with a 6 ns duration pulse and echo acquisition with 24 dB amplification by a pulser-receiver unit (5073PR, Olympus, Massachusetts, USA).

In both modalities, the signals are digitized by a data acquisition (DAQ) card (Model: M3i.4142; Spectrum Systementwicklung Microelectronic GmbH, Grosshansdorf, Germany) at a rate of 250 Megasamples per second and transferred to a personal computer (PC) for further processing and image reconstruction.

The 3D data is acquired by scanning both the detector and the illumination fiber in the x-y plane using a zig-zag scanning pattern over a field of view of 6 × 8 mm^2^, which takes ~80 sec. We use linear interpolation in the xy-plane to accommodate the data into a regular cartesian grid with 15 µm pixel size. To mitigate the effect of unwanted low frequency offsets, the measured signals are high-pass filtered with cut-off frequency at 1 MHz.

Due to the low numerical aperture and long focal depth of our light beam^27^, there is no need to adjust the optical focus during the scan, in contrast to conventional tightly focused OR-PAM systems^3,21^. The visualization methodology for the 3D optoacoustic and ultrasonic data was adopted from Ref.^29^.

### Extraction of skull geometry and elastic constants

The skull geometry extraction step (Fig. 3) is performed using cross-correlation to render a relative time delay with respect to a reference signal. We fitted a plane at each x-y location and calculated its normal **n** and the distance to the transducer. For correction of the OA data, we find the position of the first positive peak while excluding all points with the peak lying above the skull from the VCA analysis.

Both OA and US signals are acquired at a sampling frequency of 250 MHz and Fourier transformed (Fig. 3) without applying zero padding. Signal spectra are then filtered between 1–45 MHz, and 5–45 MHz for the OA and US data, respectively.

We select a single B-scan from the scanned volume and applied the VCA pipeline depicted in Fig. 3 to one particular point where the skull orientation was exactly perpendicular with respect to the focused transducer axis. A genetic algorithm optimization (Fig. 3) was then applied for 3000 iterations in order to fit a certain US reflection model to the recorded data (see more details in the supplementary information). We assumed that the geometrical effects^30^ produce the main contribution to the changes in the reflection spectrum while small variations in the thickness and elastic constants play a secondary role. Thus, the same elastic constants and skull thickness were used for the VCA correction in the region depicted in Fig. 5(b).

Note that, in calculating the IL, an uncertainty remains with respect to the actual depth of the absorbing vascular structures. Thus, we additionally run a short (maximum 12 iterations) golden-ratio optimization to extract the vasculature depth in order to optimize (minimize) the pulse-width of the deconvolved signal.

In case of low signal-to-noise ratio a Wiener filter could be formed using the IL and thus avoid the amplification of noise in the deconvolution process. As the IL is only calculated between 1 and 45 MHz, rest of the spectrum is set to zero.

The elastic constants used for the VCA corrections in phantoms and the transcranial *in vivo* experiments are listed in Tables 1 and 2. Note that values for skull 1, skull 2, skull 3, and the brain are taken from^31^ with exception of the transverse wave speed and viscous losses. As the model considers frequency dependent viscoelastic losses, the values stated for longitudinal and transverse speed of sound are those obtained without considering the viscous losses.

**Table 1:** Elastic constants used for correcting the OA signals in phantom experiments.

Layer	Fluid bottom	Skull 1	Skull 2	Skull 3	Fluid top
Thickness (µm)	Infinite	29	224	27	Infinite
Density (kg/m^3^)	1000	1969	1055	1969	1000
Longitudinal SOS (m/s)	1505	3476	1886	3476	1505
Volumetric viscosity (mPas)	2.4	10	100	10	2.4
Transverse SOS (m/s)	0	1760	650	1760	0
Shear viscosity (mPas)	1	100	1000	100	1

**Table 2:** Elastic constants used for correcting the transcranial mouse brain data.

Layer	Brain	Skull	Fluid top
Thickness (µm)	Infinite	76	Infinite
Density (kg/m^3^)	1040	1969	1000
Longitudinal SOS (m/s)	1560	3300	1488
Volumetric viscosity (mPas)	2.4	2213	2.4
Transverse SOS (m/s)	0	1560	0
Shear viscosity (mPas)	1	6573	1

### Wave propagation model

Two different types of calculations are performed in order to correct for the skull-induced distortions in the transcranial OAM data. The first one simulates the pulse-echo US measurement while the other correlates point source emissions with and without the skull. Both calculations make use of the plane wave expansion (also called angular spectrum) method in the frequency domain. Note that this requires a more comprehensive modeling approach as compared to those commonly employed in HIFU brain therapy due to (1) the very broad frequency range of the detected OA signals, as opposed to a single frequency insonication in HIFU, and (2) inclusion of time-resolved reflections in the skull model. As a result, our model can additionally simulate skull-induced reverberations. The spherically focused transducer is discretized using a frequency dependent grid and the skull is simulated as a flat isotropic multilayered solid^32^. The evaluation of the pressure field at the transducer’s surface is performed using either Fourier or Hankel series expansions, depending on the symmetry of the problem.

The pulse-echo US simulations calculate the pressure field emitted by the transducer at the skull surface using the Ring-Bessel method^33^. In order to account for an arbitrary orientation of the skull surface, the pressure field is rotated in reciprocal space^34^. The field is then weighted by the complex reflection amplitude of the solid slab and propagated back to the transducer, where the field is evaluated at its surface. A normalization spectrum, obtained from the reflection on a thick glass slab, is used to make experiments and theory directly comparable within framework of the genetic optimization (see more details in the supplementary information).

A similar procedure is employed for calculating the IL^18^. The pressure field generated by a point source is expanded as plane waves and propagated to the first fluid-solid interface, then multiplied by the complex plate’s transmission amplitude, and propagated further from the last solid-fluid interface up to the transducer’s surface, where it is evaluated. When the solid slab is not present, the field is simply propagated through free space directly onto the transducer’s surface.

The calculations are implemented in C++ and make use of CUDA GPU acceleration. A typical pulse echo calculation takes about 30 minutes (49 frequency points) for an arbitrary incidence, 5 seconds (49 frequency points) for normal incidence and 47 seconds (326 frequency points) for arbitrary orientation IL calculation on a GeForce GTX Titan X GPU, Intel Core i7 CPU 3.70 GHz.

Details of the model can be found in the supplementary information.

### Phantom experiments

Measurements of the skull IL are done using a black 20 µm diameter polyethylene microsphere (Cospheric LLC, Santa Barbara, USA). The sphere was kept in place by embedding it in an agarose block while a multimode optical fiber bundle was used to illuminate the sphere with 100 µJ laser pulses at 532 nm wavelength (see inset in Fig. 4). The acquired signals were then averaged 10000 times to increase the signal-to-noise-ratio. The skull piece was the right parietal bone of a 17 week old mouse and the experiment was performed using phosphate-buffered saline solution as the immersion medium. The approximation of the homogeneous flat solid would better fit the experimental data when the focus of the ultrasound transducer is close to the skull. Thus, the relatively long distance between the transducer focus and the skull affected the performance of the VCA results in the phantom experiments.

### In vivo experiments

A 4 weeks old female Hsd:Athymic Nude-Foxn1^nu^ mouse (Harlan Laboratories LTD, Itingen, Switzerland) was kept anesthetized with isoflurane (2.5% v∕v for induction and surgery and 2.0–2.5% v/v during imaging experiments) in 100% O_2_. The mouse head was immobilized using a custom designed stereotactic head holder (Narishige International Limited, London, United Kingdom). After scalp removal, bleeding was minimized using hemostatic sponges (Gelfoam®, Pfizer Pharmaceutical) and the skull was acoustically coupled to a Petri dish using ultrasound gel (Aquasonic clear, Parker Laboratories, Inc., Fairfield, NJ, USA). The petri dish contained distilled water and had a circular aperture at the bottom covered with a thin polyethylene foil^27^, which remained in contact with the skull and the coupling gel. *In vivo* experiments were performed in full compliance with the institutional guidelines of the Helmholtz Center Munich and with approval from the Government District of Upper Bavaria.

### Data availability

The datasets generated and analyzed during the current study are available from the corresponding author on reasonable request.

## Electronic supplementary material

Supplementary information
