# Supplementary material for: Virtual craniotomy for high-resolution optoacoustic brain microscopy
Source: Sci Rep. 2018 Jan 23;8:1459. doi: 10.1038/s41598-017-18857-y (PMC5780415; doi:10.1038/s41598-017-18857-y)
Supplement: Supplementary file 1 — Supplementary information [file 41598_2017_18857_MOESM1_ESM.pdf]

Supplementary Information
Virtual craniotomy for high-resolution optoacoustic brain microscopy

Héctor Estrada, Xiao Huang, Johannes Rebling, Michael Zwack, Sven Gottschalk, and Daniel Razansky

I. FLAT MULTILAYERED VISCOELASTIC PLATE MODEL

The skull is modelled as a multilayered viscoelastic plate of total thickness $h = \sum_{n=1}^{N} h_n = \sum_{n=1}^{3}(z_{n+1} - z_n)$ for N layers immersed between two semi-infinite fluids, which are simulated as viscoelastic solids following the global matrix method [1]. We assume plane (inhomogeneous) waves in each layer with frequency ω and $\exp(-\mathrm{i}\omega t)$ time dependence using the Stokes-Helmholtz decomposition as a solution to the Helmholtz equation. Each medium $(n = \{0, ..., N+1\})$ is isotropic and characterised by a density ρ_n, dispersion-less longitudinal $c_{\ell n}$ and transverse c_{tn} wave speed of sound, and volume ζ_n and shear η_n viscosities; all of them reflected in the complex Lamé constants $\lambda_n = \rho_n(c_{\ell n}^2 - c_{tn}^2) + \mathrm{i}\omega(2\eta_n/3 - \zeta_n)$ and $\mu_n = \rho_n c_{tn}^2 - \mathrm{i}\omega\eta_n$. With $v = \{\ell, t\}$ representing longitudinal and transverse waves, the complex bulk wavenumbers at each layer $k_{\ell n} = \omega\sqrt{\rho_n/(\lambda_n + 2\mu_n)}$, $k_{tn} = \omega\sqrt{\rho_n/\mu_n}$, and $\mathbf{k}_\parallel = \Re\{k_{vn}\}\sin(\theta_{vn})\hat{\mathbf{r}}_\parallel$ define the wavevector $\mathbf{k}_{\mathbf{z}vn} = \sqrt{k_{vn}^2 - \mathbf{k}_\parallel \cdot \mathbf{k}_\parallel}\cos(\theta_{vn})\hat{\mathbf{z}}$, with θ_{vn} being the polar angle, $\hat{\mathbf{r}}_\parallel = \cos(\varphi)\hat{\mathbf{x}} + \sin(\varphi)\hat{\mathbf{y}}$ and $\hat{\mathbf{z}}$ are unit vectors, and φ the azimuthal angle. The field can be described at each layer using a wave potential ψ_{vn} as

$$\psi_{vn} = A_{vn}^{+} e^{i\mathbf{k}_{vn}^{+}\cdot(\mathbf{r}-\mathbf{z}_{n-1})} + A_{vn}^{-} e^{i\mathbf{k}_{vn}^{-}\cdot(\mathbf{r}-\mathbf{z}_{n})}\,, \tag{1}$$

where $A_{vn}^{\pm}$, $B_{vn}^{\pm}$ are the complex amplitudes for the upwards (+) and downwards (−) longitudinal and transverse potentials. For our particular problem $A_{\ell 0}^{+} = 1$ as the incident longitudinal wave, $A_{t0}^{+} = 0$, while $R_v = A_{v0}^{-}$ are the reflected and $T_v = A_{vN+1}^{+}$ the transmitted complex amplitudes. By applying countinuity of the displacement and the stresses at the boundaries z_n one obtains a $4(N+1) \times 4(N+1)$ linear system of equations yielding $R_\ell(\mathbf{k}_\parallel, \omega)$ and $T_\ell(\mathbf{k}_\parallel, \omega)$. As we assumed that the layers are isotropic the reflection and transmission amplitudes depend only on $k_r = |\mathbf{k}_\parallel|$, i.e. $R_\ell(k_r, \omega)$ and $T_\ell(k_r, \omega)$. As we are working with inhomogeneous waves, which could return $|T_\ell| > 1$ as a solution, we implemented the test proposed in [2] based on the Deschamps pseudo-intensity [3] to rule out issues with the energy conservation. The Deschamps pseudo-intensity is in our case

$$D_{zn} = \frac{\rho_n \omega^3}{2}\left(k_{z\ell n}\psi_{\ell n}^{+}\psi_{\ell n}^{-} + k_{ztn}\psi_{tn}^{+}\psi_{tn}^{-}\right)\,, \tag{2}$$

and has to be continuous at all the z_n interfaces.

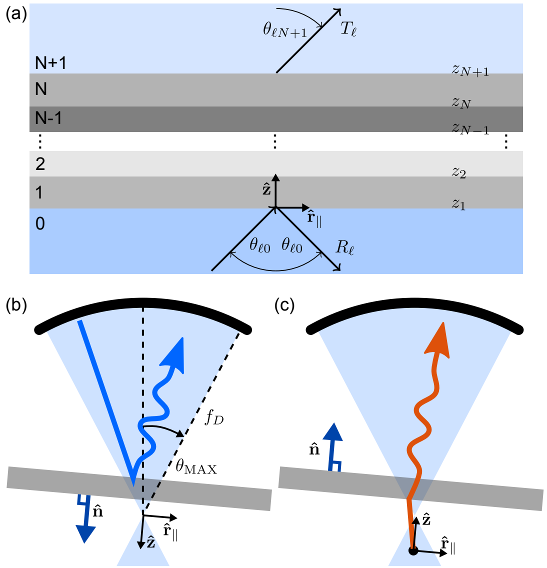

FIG. 1. (a) Diagram showing the geometry of the multilayered flat plate. Geometry of the reflection (b) and transmission (c) process

II. PLANE WAVE EXPANSION

1. Pulse-echo reflection

The geometry of the problem is illustrated in supp. Fig. 1(b). Note that the $\hat{\mathbf{z}}$ vector is pointing downwards and is tilted in order to match the orientation of the multilayered plate model. For a known incident field in medium 0 $\phi_{i0}(\mathbf{k}_\parallel, z_1, \omega) = \phi_{i0}(\omega)\phi_{i0}(\mathbf{k}_\parallel, z_1)$ in the reciprocal space and evaluated at the plate surface, the field radiated to an arbitrary plane $z < z_1$ can be written as

$$\phi_{R0}(\mathbf{r}, \omega) = \frac{\phi_{i0}(\omega)}{4\pi^2} \int_{-\infty}^{\infty} \phi_{i0}(\mathbf{k}_\parallel, z_1)\, R_\ell(\mathbf{k}_\parallel, \omega)\, \Omega_0(\mathbf{k}_\parallel, \mathbf{r})\, d^2\mathbf{k}_\parallel\,, \tag{3}$$

where $\Omega_n(\mathbf{k}_\parallel, \mathbf{r}) = \exp(\mathrm{i}\mathbf{k}_\parallel \cdot \mathbf{r}_\parallel - \mathrm{i}k_{z\ell n}(z - z_1))$. To calculate the field radiated by a spherically focused transducer we choose the Ring Bessel approach [4] due to its simplicity and speed. For a discretization of the spherically focused transducer's surface we divide the maximum transducer aperture angle θ_{MAX} into M rings of radius $R_{\parallel m}$, angle θ_m, both related by $R_{\parallel m} = f_D \sin\theta_m$, with the transducer's focal distance f_d. Each ring is at a distance z_m of the propagation plane. The incident field for a unit displacement yields

$$\phi_{i0}(k_r, z_1) = a(\omega) \sum_{n=0}^{M} \sin\theta_m \frac{e^{\mathrm{i}k_{z\ell 0} z_m}}{\mathrm{i}k_{z\ell 0}} J_0(\mathrm{k}_r R_{\parallel m})\,, \tag{4}$$

with $a(\omega) = \omega\rho_0 c_{\ell 0}(\omega) f_D^2\, \theta_{\mathrm{MAX}}/M$. If $\hat{\mathbf{n}} = \hat{\mathbf{z}}$, we can take advange of the symmetry around the azimuthal angle φ and write Eq. (3) as [5]

$$\phi_{R0}(\mathbf{r}, \omega) = \frac{\phi_{i0}(\omega)}{2\pi} \int_{0}^{\infty} \phi_{i0}(\mathrm{k}_r, z_1)\, R_\ell(\mathrm{k}_r, \omega)\, \Omega_0(\mathrm{k}_r, r, z)\, \mathrm{k}_r d\mathrm{k}_r\,, \tag{5}$$

where $\Omega_0(\mathrm{k}_r, r, z) = J_0(\mathrm{k}_r r)\exp(-\mathrm{i}k_{z\ell 0}(z - z_1))$. In the more general case where $\hat{\mathbf{n}} \neq \hat{\mathbf{z}}$, the full Eq. 3 has to be evaluated but now ϕ_{i0} in Eq. (4) represents the field radiated in a rotated coordinate system [6] and should instead be inserted back into Eq. (3) as $\phi_{i0}(\mathrm{k}'_r(\mathbf{k}_\parallel), z'_1)$, with k'_r and z'_1 defined in the rotated coordinates. Once we know the reflected field $\phi_{R0}(\mathbf{r}, \omega)$ we can evaluate it on the transducer's surface

$$\Pi_s(\omega) = \int_S \phi_{R0}(\mathbf{r}, \omega) dS = \phi_{i0}(\omega)\Gamma(\omega)\,. \tag{6}$$

We used Bessel and Fourier series to calculate Eqs. (5) and (3) respectively. Our approach is suitable to the particular spherical transducer geometry than using e.g quasi-discrete Hankel transform [7] or the fast Fourier transform, the latter is expected to be the fastest approach when modeling flat tranducers oriented parallel to the plate.

The results of this calculation, $\Pi_s(\omega)$, has to be compared directly against the pulse-echo measurements. We used $\phi_{i0}(\omega)$ as a way of normalizing the calculations. We performed pulse-echo measurements on a thick (7 mm) piece of glass and used the spectrum obtained at the focus to calculate $\phi_{i0}(\omega) = U(\omega, z = 0)/\Gamma(\omega)$, where $U(\omega, z = 0)$ represents the spectrum when the glass-water interface is at the focus. Supplementary Figure 2 shows a complete comaparison between the normalized calculation and the experimental results. The measurements have been performed at normal incidence and at different z_1, ranging from -1 to 1 mm.

2. Point source transmission

As previously described in [8], we use the plane wave expansion to calculate the Insertion Loss (IL) of a plate for a point source emitter and spherically focused detector. The field generated by a optoacoustic point source (see supp. Fig. 1(c)) can be written in frequency domain as

$$\phi_{i0}(\mathbf{r}, \omega) = \phi_{i0}(\omega)\, \frac{e^{\mathrm{i}\mathbf{k}_{\ell 0} \cdot \mathbf{r}}}{4\pi|\mathbf{r}|}\,, \tag{7}$$

which in reciprocal space at the first interface z_1 takes the form

$$\phi_{i0}(\mathbf{k}_\parallel, \omega) = \frac{\mathrm{i}\phi_{i0}(\omega)}{2}\, \frac{e^{\mathrm{i}k_{z\ell 0} z_1}}{k_{z\ell 0}}\,. \tag{8}$$

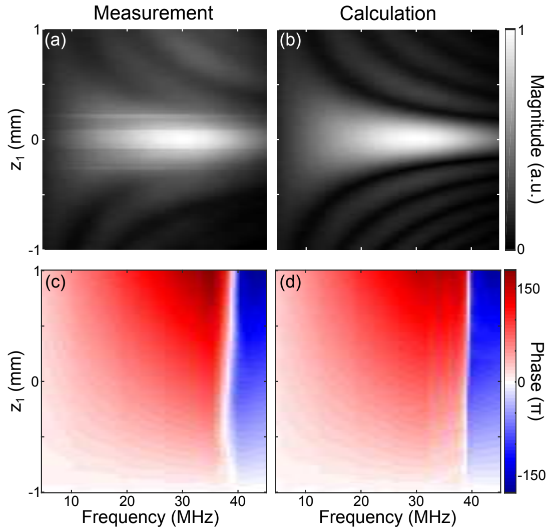

FIG. 2. Comparison between measurements (a), (c) and normalized calculations (b), (d) of the reflection spectrum of thick glass flat reflector for different depths z_1.

For $z > z_{N+1}$, one can express ϕ_{N+1} as

$$\phi_{N+1}(\mathbf{r},\omega) = \frac{\phi_{i0}(\omega)}{4\pi^2} \int_{-\infty}^{\infty} \phi_{i0}(\mathbf{k}_\parallel, z_1)\, T_\ell(\mathbf{k}_\parallel,\omega)\, \Omega_{N+1}(\mathbf{k}_\parallel,\mathbf{r})\, d^2\mathbf{k}_\parallel\,. \tag{9}$$

Due to the symmetry in φ, we can write [5]

$$\phi_{N+1}(\mathbf{r},\omega) = \frac{\phi_{i0}(\omega)}{2\pi} \int_0^{\infty} \phi_{i0}(\mathrm{k}_r, z_1)\, T_\ell(\mathrm{k}_r,\omega)\, \Omega_{N+1}(\mathrm{k}_r, r, z)\, \mathrm{k}_r d\mathrm{k}_r\,. \tag{10}$$

As the source is a sphere, the symmetry is always preserved, regardless of the plate orientation, provided the solids are isotropic. Therefore, Eq. (10) can always be used, even with multiple point sources if the source distribution maintains the symmetry. Without the plate, Eq. (10) takes the form

$$\phi_{N+1}(\mathbf{r},\omega) = \mathrm{i}\frac{\phi_{i0}(\omega)}{4\pi} \int_0^{\infty} \frac{\Omega_{N+1}(\mathrm{k}_r, r, z)}{k_{z\ell N+1}}\, \mathrm{k}_r d\mathrm{k}_r\,. \tag{11}$$

Again, due to the geometry of the transducer, the Bessel series expansion yields the fastest calculation. We used a maximum of 1950 radial spectral points for the maximum frequency, 45 MHz, and adjusted the number of points for lower frequencies to speed up the calculations according to Eq. (22) in [9] down to a minimum of 100 points for low frequencies ($<$ 5 MHz). Finally, in order to calculate the insertion loss we introduce

$$\Pi_0(\omega) = \int_S \phi_{i0}(\mathbf{r},\omega) dS\,, \tag{12}$$

which is proportional to the pressure detected by the transducer without the plate and

$$\Pi_s(\omega) = \int_S \phi_{N+1}(\mathbf{r},\omega) dS\,, \tag{13}$$

when the is in the wave propagation path. Then we compute the insertion loss as

$$\mathrm{IL}(\omega) = \frac{\Pi_0(\omega)}{\Pi_s(\omega)}\,. \tag{14}$$

3. Deconvolution

If the signal-to-noise ratio of the original dataset is high enough, one could attempt direct deconvolution using $\mathrm{IL}(\omega)$ as

$$V(\omega) = P(\omega)\,\mathrm{IL}(\omega)\,, \tag{15}$$

where $P(\omega) = \mathcal{F}_t\left\{p(t)\right\}$ is the original signal in frequency domain. The corrected signal is then $v(t) = \mathcal{F}_\omega^{-1}\left\{V(\omega)\right\}$. On the other hand, if the signal-to-noise ratio in the original data set is low, the direct deconvolution by $\mathrm{IL}(\omega)$ might result in unwanted noise amplification. A Wiener filter can be constructed from the IL using the insertion gain $\mathrm{IG}(\omega) = \mathrm{IL}(\omega)^{-1}$ to avoid noise amplification as

$$W(\omega) = \frac{\mathrm{IG}(\omega)^*}{|\mathrm{IG}(\omega)|^2 + \frac{1}{\mathrm{SNR}(\omega)}}\,. \tag{16}$$

We can deconvolve

$$V(\omega) = P(\omega)\,W(\omega)\,, \tag{17}$$

and finally obtain the deconvolved signal

$$v(t) = \mathcal{F}_\omega^{-1}\left\{V(\omega)\right\}\,. \tag{18}$$

III. OPTIMIZATION

4. Genetic algorithm for pulse-echo ultrasound

The genetic optimization algorithm was previously implemented in [8] and has been now modified to deal with pulse-echo data instead of the insertion loss. The previous implementation only optimized the magnitude of the IL, whereas this new implementation optimizes both, magnitude and phase of the pulse-echo frequency spectrum.

Our implementation is semi-automated due to the large size of the parameter space and the imperfect fit of our model, which does not consider the porosity of the bone.

The core of the genetic algorithm performs the following steps:

1. An initial population of plates is generated by random sampling of the parameter space within certain specified limits. One individual of the population is the first guess, that requires a manual input of all the parameters.

2. A population of N individuals is evaluated and ranked based on a minimum of the cost function. We used the cost function

$$\mathrm{CF_n} = \int_{f_1}^{f_2} |\Pi_{s,n}(f) - \Pi_e(f)|^2\,df\,, \tag{19}$$

   where f is the frequency, $\Pi_{s,n}(f)$ is defined in Eq. (6) for the n-plate of the population, and $\Pi_e(f)$ is the pulse-echo measurement.

3. A new generation of J plates is obtained by breeding random plates of the population with bias depending on their rank. The breeding is performed using a random parameter mask M such that $\mathrm{plate}_3 = M\mathrm{plate}_1 + (1-M)\mathrm{plate}_2$. Random mutations are introduced in the new offspring after breeding. The number of the mutated parameters is a function of the generation and is chosen to decrease as the population evolves.

4. Each new generation is evaluated and ranked. The plates in the population with worst rank are discarded and the previous point is repeated until the maximum number of algorithm iterations is reached.

In addition to the initial guess, the selection of the range of the parameter space is key for achieving satisfactory results. We performed several runs in order to optimize different parameters depending on their relevance. From our experience, h, $c_{\ell 2}$, c_{t2} constitute the more important parameters, while ρ_2, χ, η are secondary. As a first step, it is helpful to look at distinctive feature in the time domain signal. If the two boundaries of the skull can be distiguished (even if it isn't clear), the time of flight t_f and an upper bound for $c_{\ell 2} < 4000$ (m/s) can be used to constrain the thickness range.

A general optimization procedure can be summarized as:

1. Run a first optimization only for h within the range found with help of the time domain signal and parameters found in the literature.

2. Run a second optimization round changing h within a smaller range and linking $c_{\ell 2} = h/t_f$.

3. Search now for h and $c_{\ell 2}$ simultaneously in a tighter range.

4. Refine the optimization for c_{t2}.

5. Refine the optimization for η.

6. Now refine within very tight limits (±3% variation) for all the parameters including some of the best results of previous runs in the initial population.

7. Repeat the procedure for different convergence parameters of the genetic algorithm and/or refine even more the limits of the parameter space.

5. *Golden ratio optimization of* z_1

The cost function used in the golden ratio optimization is the inverse of the width of the deconvolved pulse. The pulse width is measured as the maximum distance between point that are above the 10% of relative amplitude. In order to rule out an unexpected behavior of the algorithm, we explored the deconvolved pulse-width as a function of the source depth and observed the existence of a few local maxima that could yield an inaccurate value. The behavior was consistent for a few cases and we established a rule of thumb for the method to converge to the global maximum provided that a) the source cannot be inside the skull and b) it is very unlikely it will go deeper than 1 mm below the skull due to constrains in the light propagation.

---

[1] M. Lowe, Ultrasonics, Ferroelectrics, and Frequency Control, IEEE Transactions on **42**, 525 (1995).
[2] M. A. Ainslie and P. W. Burns, The Journal of the Acoustical Society of America **98**, 2836 (1995).
[3] M. Deschamps, Journal dAcoustique **3**, 251 (1990).
[4] U. Vyas and D. A. Christensen, The Journal of the Acoustical Society of America **130**, 2687 (2011).
[5] L. M. Brekhovskikh and O. A. Godin, *Acoustics of Layered Media*, 2nd ed., Springer Series on Wave Phenomena, Vol. II (Springer-Verlag Berlin Heidelberg, 1999).
[6] G. Clement and K. Hynynen, Ultrasonics, Ferroelectrics, and Frequency Control, IEEE Transactions on **50**, 1689 (2003).
[7] M. Guizar-Sicairos and J. C. Gutiérrez-Vega, J. Opt. Soc. Am. A **21**, 53 (2004).
[8] H. Estrada, J. Rebling, J. Turner, and D. Razansky, Physics in Medicine and Biology **61**, 1932 (2016).
[9] F. Shen and A. Wang, Appl. Opt. **45**, 1102 (2006).
